# A prospective randomized controlled clinical trial investigating the efficacy of low-dose olanzapine in preventing nausea and vomiting associated with oxaliplatin-based and irinotecan-based chemotherapy

**DOI:** 10.1007/s00432-024-05712-7

**Published:** 2024-05-28

**Authors:** Jing Shen, Juan Zhao, Gaowa Jin, Hui Li, Ying Jiang, Yungaowa Wu, Jiali Gao, Feng Chen, Jiaxuan Li, Wenjuan Wang, Quanfu Li

**Affiliations:** 1 Ordos Clinical College, Baotou Medical College, Ordos, 017000 China; 2 Department of Medical Oncology, Ordos Central Hospital, 23th Yijinhuoluo Western Road, Dongsheng District, Ordos, 017000 China; 3 Ordos Clinical College, Inner Mongolia Medical University, Ordos, 017000 China

**Keywords:** CINV, Entire age bracket, Female, Low-dose olanzapine, Moderately emetogenic chemotherapy

## Abstract

**Objective:**

The aim of this study is to assess the clinical efficacy of a 5 mg dosage of olanzapine in preventing chemotherapy-induced nausea and vomiting (CINV) associated with moderately emetogenic chemotherapy (MEC) among female patients diagnosed with gastrointestinal tract tumors.

**Methods:**

Patients undergoing the oxaliplatin/irinotecan chemotherapy regimen were enrolled in this prospective controlled study. The olanzapine group received a 5 mg dosage of olanzapine along with palonosetron and dexamethasone, while the control group received a standard two-combination regimen consisting of dexamethasone and palonosetron. The primary endpoints included the total protection (TP) rates for the entire age group and the subgroup aged 60 years and above. Secondary endpoints encompassed the total protection rates during the acute and delayed phases within the two age brackets, as well as the total control (TC) rates and complete remission (CR) rates across all three phases (total, acute, and delayed). Additionally, the study involved the assessment of quality of life and the collection of adverse events associated with the interventions.

**Results:**

1) Regarding the primary endpoint, the total phase TP rates within both the entire age group and the age group exceeding 60 years demonstrated superiority in the olanzapine group when compared to the control group (66.7% vs 37.25%, *P* = 0.003; 68.8% vs 44.4%, *P* = 0.044). 2) In terms of secondary endpoints, the olanzapine group exhibited superior acute phase TP rates in both age brackets when compared to the control group (*P* < 0.05). The olanzapine group also demonstrated higher delayed-phase TP rates, TC rates across all three phases, and CR rates within the two age brackets, although the differences were not statistically significant (*P* > 0.05). Furthermore, the quality of life in the olanzapine group surpassed that of the control group for both age brackets (*P* < 0.05), characterized by enhanced appetite and a higher incidence of drowsiness in the patients treated with olanzapine when compared to those in the control group (*P* < 0.05).

**Conclusion:**

Olanzapine can enhance CINV induced by MEC regimen in female patients across all age groups, including the elderly, and therefore improve the quality of life for these patients.

**Clinical Trial Registration:** https://www.chictr.org.cn/index.html, identifier: ChiCTR20000368269, 25/08/2020.

## Introduction

Chemotherapy-induced nausea and vomiting (CINV) constitutes a prevalent adverse reaction to chemotherapy, and its severity leads to delayed administration of chemotherapy, diminished relative dose intensity, and notably abbreviated disease-free survival (DFS) and overall survival (OS) for patients (Woopen et al. 2020). Even among patients with high-risk factors for CINV, such as young females, non-alcohol consumers, and those with anxiety, approximately 48% still encounter nausea and vomiting despite receiving standard antiemetic prophylaxis regimens (Sekine et al. 2013; Hesketh et al. 2006). XELOX (capecitabine + oxaliplatin) or SOX (S-1/Oxaliplatin) is recommended as a postoperative adjuvant regimen or as an initial-grade I chemotherapy regimen (Yu et al. 2019; Zhang et al. 2021). For advanced colorectal cancer, XELIRI (capecitabine + irinotecan) or FOLFIRI (5-fluorouracil/calcium folinate/irinotecan) serves as the standard first- and second-line chemotherapy regimens (Wu et al. 2020bemetogenic chemother; Cremolini et al. 2020). Prior studies indicate that, in the absence of preventive measures, the incidence of nausea and vomiting resulting from the Folinic acid, fluorouracil and oxaliplatin (FOLFOX) regimen as an adjuvant chemotherapeutic approach was 73.7 and 47.2%, respectively (Andre et al. 2004). Meanwhile, the incidences of nausea and vomiting attributed to the FOLFIRI regimen ranged from 48 to 62% and 24 to 32%, respectively (Shimada et al. 1993)..

Based on these findings, oxaliplatin or irinotecan-based chemotherapy regimens exhibit a substantial potential to induce CINV, with the likelihood of inducing nausea being significantly higher than that of vomiting. In a multicenter, randomized, open-label, and phase III study conducted in Japan to compare the efficacy of a three-drug combination regimen incorporating a neurokinin-1 receptor antagonist (NK1 RA) with that of a standard two-drug combination regimen in patients diagnosed with colorectal cancer undergoing oxaliplatin-based chemotherapy, the results indicated that the proportions of patients free from vomiting in the total phase, acute phase, and delayed phase in the aprepitant group were significantly higher than those in the control group (95.7 vs 83.6%, *P* = 0.0001; 100 vs 96.7%, *P* = 0.013; 95.7 vs 84.7%, *P* = 0.0003) (SNishimura J et al. 2015). However, another double-blind, placebo-controlled study assessing the preventive effect of NK-1RA on oxaliplatin-induced CINV yielded inconsistent conclusions, revealing no significant differences in the CR rates between the NK-1RA group and the control group in the total phase (86% vs 85%), the acute phase (97% vs 96%), and the delayed phase (86% vs 85%) (*P* > 0.05) (Hesketh et al. 2012). Based on these two studies, the Multinational Association of Supportive Care in Cancer/European Society for Medical Oncology (MASCC/ESMO) guidelines do not recommend the use of NK1 RA in combination for the prevention of oxaliplatin-induced CINV. In contrast, the National Comprehensive Cancer Network (NCCN) guidelines recommend the combination with NK-1 RA, considering oxaliplatin and irinotecan as high emetogenic risk factors for CINV in certain patient populations. These two international guidelines present inconsistent perspectives on the role of NK1RA in moderately emetogenic chemotherapy (Razvi et al. 2019).

A controlled study comparing 5 mg olanzapine to aprepitant in the prevention of CINV induced by chemotherapy regimens with a high emetogenic risk demonstrated comparable efficacy between the two, with 5 mg olanzapine exhibiting a superior advantage in managing nausea and significantly enhancing the quality of life of the patients (Liu et al. 2022). Despite a limited number of prospective clinical studies assessing the antiemetic effect of olanzapine in MEC, these studies suffered from small sample sizes and lacked stratification based on tumor groups and chemotherapy regimens (Navari et al. 2005; Tan et al. 2009; Mizukami et al. 2014; Jeon et al. 2019). Furthermore, some studies amalgamated severe and moderate emetogenic regimens.

A 2020 retrospective study conducted by Wu confirmed the enhanced efficacy of a triple-drug antiemetic regimen in controlling CINV among patients diagnosed with gastrointestinal cancer having high emetogenic risk factors receiving MEC regimens (mFOLFOX6/FOLFIRI/XELOX) (Wu et al. 2020a). However, the study exclusively included female patients under the age of 55 with minimal or no alcohol consumption, raising uncertainty about the generalizability of these findings to patients of all ages. Therefore, it becomes imperative to conduct a comprehensive study on the efficacy of 5 mg olanzapine in combination with 5-HT3 receptor antagonist palonosetron and dexamethasone for preventing CINV in a diverse age group of female patients with gastrointestinal tumors undergoing a MEC regimen.

## Materials and methods

### General information

In this prospective randomized controlled clinical study, female patients with gastrointestinal tumors undergoing MEC (oxaliplatin/irinotecan) were selected and enrolled utilizing the randomized numeric table method. The general characteristics, such as age, disease type, and chemotherapy regimen, among others, were recorded. Tables 1, 2 displays the baseline characteristics. The FILE scale was used to record the frequency and severity of nausea and vomiting occurring within the 0–120 h timeframe post-chemotherapy for the selected female patients with gastrointestinal tumors receiving MEC (oxaliplatin/irinotecan). Additionally, the type and severity of concomitant adverse effects were similarly documented.

**Table 1:** Baseline data of patients of the entire age group

Patient characteristics	Olanzapine group (*n* = 51)	Control group (*n* = 51)	*P*
Age	60.43 ± 9.366	62.33 ± 9.911	0.322
Type of malignnce			1.000
Gastric cancer	12 (23.6%)	17 (33.3%)	
Colon cancer	7 (13.7%)	10 (19.6%)	
Rectal cancer	32 (62.7%)	24 (47.1%)	
Chemotherapy regimen			0.979
XELOX	33 (64.7%)	29 (56.9%)	
SOX	10 (19.6%)	9 (17.6%)	
FOLFOX	5 (9.8%)	10 (19.6%)	
FOLFIRI	2 (3.9%)	3 (5.9%)	
XELIRI	1 (2.0%)	0 (0%)

**Table 2:** Baseline of patients of the age group over 60 years

Patient characteristics	Olanzapine group (*n* = 32)	Control group (*n* = 36)	*P*
Age	66.44 ± 4.655	67.611 ± 5.095	0.327
Type of malignnce			0.844
Gastric cancer	9 (28.1%)	16 (44.4%)	
Colon cancer	2 (6.2%)	6 (16.7%)	
Rectal cancer	21 (65.6%)	14 (38.9%)	
Chemotherapy regimen			0.839
XELOX	21 (65.6%)	19 (52.8%)	
SOX	8 (25.0%)	9 (25.0%)	
FOLFOX	2 (6.2%)	5 (13.9%)	
FOLFIRI	1 (3.1%)	3 (8.3%)	

### Inclusion criteria

(1) Pathologically diagnosed as gastrointestinal malignant tumors.

(2) Female, aged between 18 and 80 years old.

(3) Prior to chemotherapy, there were no irregularities observed in the electrocardiogram, complete blood count, liver function, or kidney function. The neutrophil count exceeded 1.5 × 10^9^/L, the white blood cell count surpassed 3.5 × 10^9^/L, and the platelet count surpassed 85 × 10^9^/L. The alanine aminotransferase, alkaline phosphatase, and bilirubin levels were all below 2.5 × 10^9^/L, respectively, and the upper limit of the normal was not exceeded.

(4) No history of alcohol consumption.

(5) No nausea or vomiting symptoms 1 week before enrollment, and no application of aprepitant or olanzapine.

(6) Agreed to receive a triple-drug regimen of olanzapine combined with palonosetron and dexamethasone or a double-drug regimen of palonosetron and dexamethasone.

(7) Informed signed consent.

### Exclusion criteria

(1) Unable to take oral medications.

(2) Patients who are experiencing nausea or vomiting for reasons unrelated to chemotherapy, such as gastrointestinal blockage, ascites necessitating puncture, brain metastases, or other brain tumors/lesions.

(3) Unable to receive standard dose dexamethasone due to uncontrolled diabetes or gastrointestinal bleeding, etc.

(4) Being treated with other antipsychotics or NK1 receptor antagonists.

(5) Serious underlying diseases such as severe heart disease, and liver and kidney disease.

(6) During pregnancy or lactation.

(7) Unable to complete the FLIE scale assessment.

### Study protocol

In both groups, intravenous injections of dexamethasone 10 mg and panolosetron 0.25 mg were administered 30 min before chemotherapy. The olanzapine group received an oral dose of 5 mg olanzapine nightly for a continuous period of 5 days, commencing on the day of chemotherapy (Table 3).

**Table 3:** Prevention program

	Day1	Day2	Day3	Day4	Day5
Olanzapine group	10 mg dexamethasone Intravenous drip				
	0.25 mg palonosetron Intravenous drip				
	5 mg olanzapine Oral	5 mg olanzapine Oral	5 mg olanzapine Oral	5 mg olanzapine Oral	5 mg olanzapine Oral
Control group	10 mg dexamethasone Intravenous drip				
	0.25 mg palonosetron Intravenous drip				

### Efficacy assessment

During the 0–120 h post-chemotherapy, the study aimed to document the frequency, duration, and severity of daily occurrences of nausea and vomiting. Nausea was assessed using the FILE scale for both the acute phase (0–24 h) and the delayed phase (24–120 h) of post-chemotherapy nausea. The primary endpoint indicators were the TP rates encompassing the entire age bracket and the age bracket exceeding 60 years during the total phase (acute phase + delayed phase or 0–120 h after chemotherapy). TP was defined as the absence of vomiting or severe retching without the need for treatment measures, accompanied by a maximum nausea score of ≤ 25 mm on a 100 mm nausea assessment scale. Secondary endpoints included TP rates for the acute phase (0–24 h) and delayed phase (25–120 h) within the two age brackets, as well as TC rates and CR rates across all three phases (total phase, acute phase, and delayed phase). The TC is defined as no vomiting or severe retching requiring no treatment measures, with a maximum nausea score of ≤ 5 mm on a 100 mm nausea assessment scale. Quality of life was assessed using the FILE questionnaire, with no impact on daily life defined when the total FILE score was > 108 (Decker et al. 2006; Martin et al. 2003). Adverse events related to the antiemetic drugs in both groups, such as constipation, drowsiness, and loss of appetite, were diligently recorded.

## Data statistics

The entirety of the data underwent statistical analysis using SPSS 26.0 software. Measurement data that adhered to a normal distribution are expressed as mean ± standard deviation. The comparison within groups employed the paired *t*-test, while the independent *t*-test was used for the comparison between groups. The comparison of count data was conducted using the chi-squared test. A statistical analysis difference was deemed statistically significant when the p-value was less than 0.05.

The hypothesis of this study is that the TP rate of olanzapine will be statistically significantly higher than that of the control group. Previous trials have shown that standard dual antiemetic therapy has a TP rate of approximately 40% (Wu et al. 2020). We estimate that an increase in TP rate of > 15% would be meaningful. Therefore, the invalid hypothesis to speculate on the TP rate is 55% and the other hypothesis is 80%, and we calculate that it would take at least 90 patients to achieve a one-sided type I error of 0.1% and a power of 80%. Since some people were expected to drop out, we set a target sample size of 100 and the sample size calculation was done by SASV.9.4 (Cary, NC, USA).

## Results

**Fig. 1:**
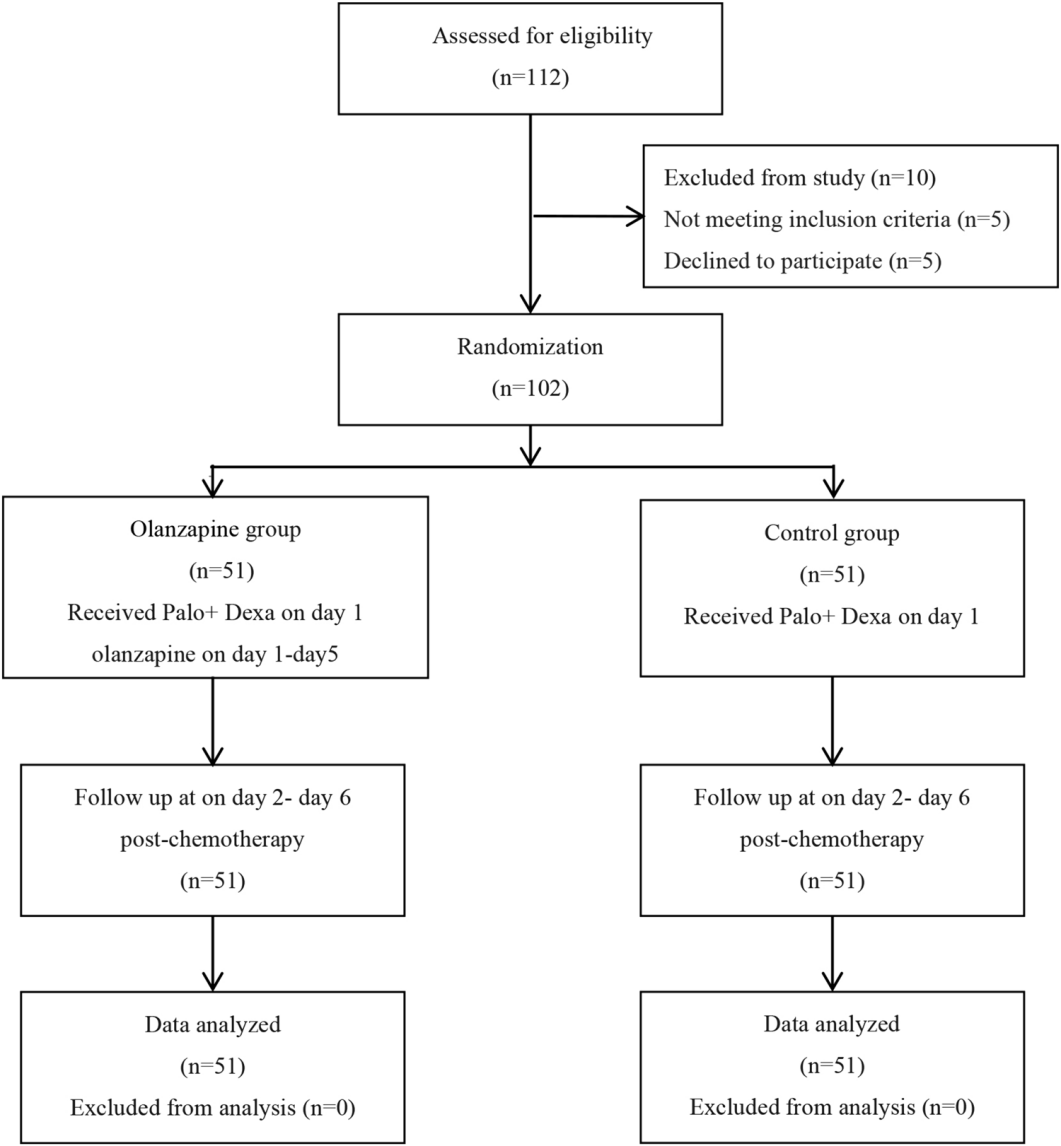
Patient screening and selection

A total of 102 participants successfully concluded the follow-up assessments for primary and secondary endpoint parameters. The distribution and randomization of patients was displayed in Fig. 1. This group comprised of 51 patients allocated to the olanzapine group and an equivalent number assigned to the control group, all of whom completed the follow-up assessments across the entire age spectrum. Within the subgroup aged 60 years and above, 68 patients underwent the follow-up procedure, with 32 belonging to the olanzapine group and 36 to the control group.

### Primary endpoint indicators

**Fig. 2:**
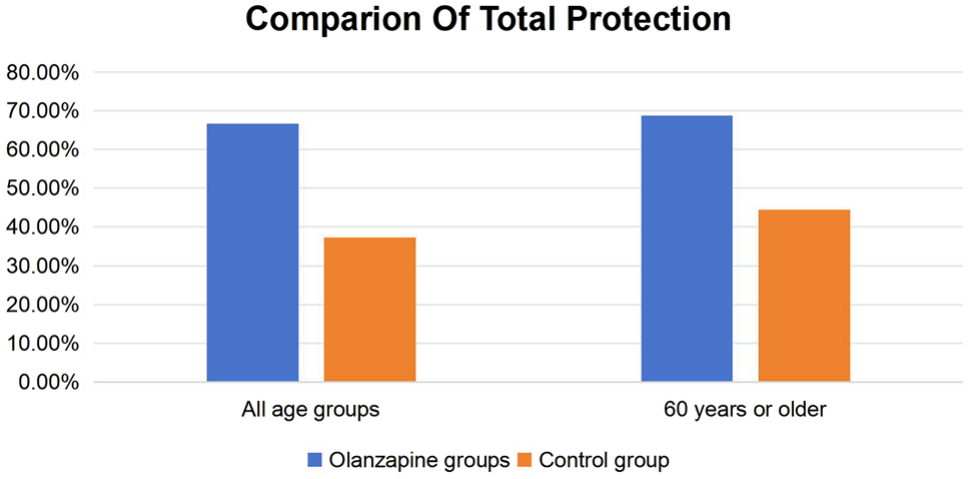
Comparison of the primary endpoint indicator TP rate between the two age groups

The total TP rates for the overall age group within the olanzapine and control groups were 66.7 and 37.25%, respectively, demonstrating a statistically significant difference (*P* = 0.003). Specifically, within the subset of patients aged 60 years and above, the TP rates were 68.8 and 44.4% for the olanzapine and control groups, respectively, with a statistically significant distinction (*P* = 0.044) (refer to Fig. 2).

### Secondary endpoint indicators

The acute phase TP rates for both the overall age group and the subset age bracket over 60 years and above were higher in the olanzapine group when compared to the control group, with statistically significant differences observed (*P* = 0.025, *P* = 0.034, respectively). Conversely, there were no statistically significant differences in the TN rate and CR rate between the two age brackets across all phases (*P* > 0.05) (refer to Tables 4, 5). The quality of life in both age brackets demonstrated a significant enhancement in the olanzapine group as opposed to the control group (FILE scale > 108 points) (84.31% vs 58.80%, *P* = 0.003; 84.38% vs 58.33%, *P* = 0.041) (see Tables 6, 7).

**Table 4:** Indicators of secondary endpoints for the entire age group

	Olanzapine group	Control group	*P*
All age groups TP			
Acute phase	72.50%	51.00%	0.025
Delay phase	68.60%	52.90%	0.106
All age groups TC			
Acute phase	41.20%	29.40%	0.214
Delay phase	37.20%	35.30%	0.837
Overall phase	31.40%	21.60%	0.262
All age groups CR			
Acute phase	84.30%	78.40%	0.445
Delay phase	80.30%	76.50%	0.630
Overall phase	76.50%	66.70%	0.272

**Table 5:** Secondary endpoint indicators for patients of the age group of over 60 years

	Olanzapine group	Control group	*P*
60 years or older groups TP			
Acute phase	75.00%	50.00%	0.034
Delay phase	71.90%	63.90%	0.482
60 years or older groups TC			
Acute phase	43.80%	30.60%	0.260
Delay phase	37.50%	38.90%	0.906
Overall phase	28.10%	25.00%	0.771
60 years or older groups CR			
Acute phase	84.40%	80.60%	0.680
Delay phase	75.00%	75.00%	1.00
Overall phase	71.90%	63.90%	0.482

**Table 6:** Comparison of FLIE index of the entire age group between the two groups

Iems	Olanzapine group	Control group	*P*
Nausea FLIE Score	54.90 ± 10.91	48.56 ± 13.04	0.009
Vomiting FLIE Score	60.01 ± 11.37	57.27 ± 12.23	0.198
FLIE Score	114.46 ± 18.57	107.51 ± 20.42	0.075

**Table 7:** Comparison of FLIE index of the age group over 60 years between the two groups

Items	Olanzapine group	Control group	*P*
Nausea FLIE Score	55.53 ± 10.92	50.86 ± 11.37	0.089
Vomiting FLIE Score	60.07 ± 7.73	57.77 ± 9.85	0.291
FLIE Score	114.89 ± 17.44	107.96 ± 18.45	0.118

## Assessment of adverse reactions

The predominant adverse reaction observed in the olanzapine group was drowsiness, occurring at a rate of 52.94% (27/51), while the control group exhibited a lower incidence at 31.37% (16/51), indicating a statistically significant disparity (*P* = 0.027). Conversely, the olanzapine group demonstrated a reduced occurrence of loss of appetite when compared to the control group (56.86% vs 72.55%, *P* = 0.097). Notably, olanzapine contributed to an enhancement in appetite, with a frequency of 43.14%, as opposed to 27.45% in the control group, reaching statistical significance (*P* = 0.036). Patients exhibited tolerance to the adverse effects of the treatment, and notably, no grade 3–4 adverse effects were reported. The incidences of adverse reactions are delineated in Table 8.

**Table 8:** Incidences of adverse reactions

Adverse reactions	Olanzapine group (%)	Control group (%)	*P*
Hiccup	21.57	33.33	0.183
Constipation	15.69	27.45	0.149
Weakness	78.43	92.16	0.050
Dizziness	43.14	39.22	0.687
Headache	21.57	37.25	0.082
Drowsiness	52.94	31.37	0.027
Loss of appetite	56.86	72.55	0.097
Upper abdominal bloating	19.60	31.37	0.173

## Discussion

Two extensive phase III clinical trials have previously conducted a comparative assessment of the efficacy of a two-drug regimen combined with NK1 RA against a standard two-drug regimen in patients diagnosed with colorectal cancer undergoing oxaliplatin-based chemotherapy (SNishimura J et al. 2015; Hesketh et al. 2012). However, the outcomes of these two trials were inconsistent. The question of whether the addition of NK-1 RA is advisable for the prevention of CINV in patients undergoing moderately emetogenic chemotherapy remains contentious. Consequently, the MASCC/ESMO and NCCN guidelines currently lack consensus on the role of NK1RA in oxaliplatin-based chemotherapy. In recent years, a phase III, double-blind, placebo-controlled study conducted by Sun Yat-sen University assessed aprepitant along with a standard antiemetic regimen for preventing CINV induced by MEC in female patients under the age of 50 with high-risk gastrointestinal tumors and minimal or no alcohol consumption. This study demonstrated a heightened antiemetic efficacy of aprepitant when combined with palonosetron and dexamethasone within the context of FOLFOX or FOLFIRI chemotherapy regimens (Wang et al. 2021). A controlled study investigating the prevention of CINV induced by chemotherapy regimens with a high emetogenic risk revealed comparable efficacy between olanzapine and aprepitant, with olanzapine exhibiting superiority in controlling nausea (Liu et al. 2022). Furthermore, in a retrospective clinical study led by Professor Wu, which encompassed female patients under the age of 55 with minimal or no alcohol consumption and high-risk gastrointestinal tumors, the results indicated that the CR rates during both the delayed and total phases of a triple antiemetic regimen, consisting of 5 mg olanzapine for preventing MEC-induced CINV, were higher in the olanzapine group than in the control group (75.0% vs 54.7%, *P* = 0.044; 70.0% vs 47.2%, *P* = 0.028). Additionally, the olanzapine group exhibited superior efficacy in controlling nausea during the total phase (62.5% vs 39.6%, *P* = 0.029) (Wu et al. 2020).

Both of the aforementioned studies have affirmed the efficacy of triple antiemetic regimens comprising of olanzapine or aprepitant in effectively managing CINV in patients with gastrointestinal tumors undergoing MEC regimens such as FOLFOX6/FOLFIRI. These regimens have demonstrated efficacy particularly in patients with high risk factors for vomiting, including females below 55 or 50 years of age and those with minimal or no alcohol consumption. In alignment with this evidence, the current prospective randomized controlled clinical study aimed to assess the prevention of CINV induced by MEC through the administration of a triple antiemetic regimen consisting of 5 mg olanzapine, 5-HT3 receptor antagonist (RA), and dexamethasone in female patients of all ages with gastrointestinal tumors. The primary study endpoints were successfully achieved. Notably, the overall TP rates for the entire age group in the olanzapine group and the control group were 66.7 and 37.25%, respectively (*P* = 0.003). Moreover, the TP rates for the subgroup aged over 60 years were 68.8% vs 44.4% (*P* = 0.044), indicating a statistically significant difference. The study substantiates the clinical efficacy of 5 mg olanzapine in preventing CINV induced by MEC, particularly in ameliorating nausea. These findings align with the outcomes of a phase III clinical trial conducted by Tan et al., which involved 229 patients undergoing moderate or high emetogenic chemotherapy regimens. This trial demonstrated that patients receiving MEC regimens, along with a standard antiemetic regimen (5-HT3 + dexamethasone) in combination with 10 mg olanzapine, exhibited higher CR rates for both nausea and vomiting compared to the control group (83.07% vs 56.45%, *P* < 0.05; 89.23% vs 75.80%, *P* < 0.05) (Tan et al. 2009 Sep 23). Although the study did not use TP rates as study indexes, the statistically significant enhancement in the CR rate of nausea in the olanzapine group further supports the conclusion that the addition of 5 mg olanzapine significantly enhances nausea control.

Due to the potent sedative effects associated with a 10 mg dosage of olanzapine, a substantial 73% of patients receiving this dose experienced drowsiness. Prior clinical studies have established comparable efficacy between 5 and 10 mg olanzapine in CINV prevention, with the former exhibiting a lower incidence of drowsiness (Tan et al. 2009; Yanai et al. 2018). This provides a rationale for employing a 5 mg olanzapine dosage in the current study protocol. Nausea presents a significant challenge in CINV management, and olanzapine is particularly advantageous in mitigating chemotherapy-induced nausea (Roscoe et al. 2010). As the primary endpoint indicators, the TP rate, which focuses on nausea assessment, was used. The total TP rate in the olanzapine group, when compared to the control group, was 66.7% vs. 37.25% (*P* = 0.003), displaying statistical significance and consistent findings with the study conducted by Wu on the total TP rate (62.5% vs. 39.6%, *P* = 0.029) (Wu et al. 2020a). In terms of CR rates, a comparison with the study conducted by Wu reveals closely aligned results. The CR rates in the olanzapine group for the total, acute, and delayed phases were 70.0% vs 76.50%, 85.0% vs 84.30%, and 75.0% vs 80.30%, respectively. Although statistically insignificant differences were observed in CR rates between the two groups in the present study, this may be attributed to higher CR rates in the control group. Potential reasons for this discrepancy include the retrospective nature of the study conducted by Wu, possibly leading to an underestimation of CINV incidence and severity in the control group. In contrast, the present study is prospective. Additionally, the use of the second-generation 5-HT3 receptor antagonist palonosetron in the present study, as opposed to the first-generation drug in the study conducted by Wu, may contribute to the lack of statistical significance. Palonosetron, known for its enhanced efficacy and safety, boasts a 30- to 100-fold higher affinity for 5-HT3 receptors than its predecessor, with a prolonged average terminal elimination half-life of up to 40 h. This extended half-life ensures sustained action into the delayed phase in the control group (Aapro 2006; Schwartzberg et al. 2014).

In a phase III double-blind, placebo-controlled study conducted by Wang from Sun Yat-sen University in 2021, which included patients with gastrointestinal tumors and high-risk factors for vomiting (females less than 55 or 50 years, little or no alcohol consumption) undergoing MEC, the total TP rate in the aprepitant group when compared to the control group (87.0% vs 66.7%; *P* < 0.001) exhibited a similarity to the current study (66.7% vs 37.25%; *P* = 0.003) (Wang et al. 2021). Furthermore, the CR rates for the total, acute, and delayed phases in the aprepitant group when compared to the control group (87.0% vs 66.7%; *P* < 0.001; 92.7% vs 75.8%; *P* = 0.001; 88.6% vs 70.0%; *P* = 0.001) were contrasted with the CR rates of the present study (76.50% vs 66.70%, *P* = 0.272; 84.30% vs 78.40%, *P* = 0.445; 80.30% vs 76.50%, *P* = 0.630). Notably, while the CR rates were comparable in the control group of the two studies, the experimental group in the current study exhibited lower CR rates compared to the experimental group in the study conducted by the Sun Yat-sen University. Several factors may contribute to these variations. Firstly, the regional hospital where participants were recruited in the present study catered to a population with advanced gastrointestinal tract tumors undergoing second-line treatment after disease progression with first-line medication. This subgroup, characterized by lower body mass scores, could impact the efficacy of CINV treatment. Additionally, a subgroup analysis of the Sun Yat-sen University study focused on preventing oxaliplatin-containing regimens for CINV. In this subgroup, the aprepitant group demonstrated a significantly higher total CR rate compared to the control group (89.8% vs. 66.3%; *P* < 0.001). In contrast, among the participants in the present study, 94.1% received oxaliplatin-containing chemotherapy regimens, and the total CR rate in the olanzapine group was higher than that in the control group (77.0% vs 68.75%; *P* = 0.358), although statistically insignificant. It is important to note that the Sun Yat-sen University study had specific inclusion criteria, including only female patients under 50 years old with minimal or no alcohol consumption, limiting the generalizability of its results to a broader clinical context. However, it holds higher clinical utility value (Qi et al. 2023). The findings of the current study align with those of a randomized controlled clinical study by Jeon in South Korea in 2019, which investigated a triple regimen including olanzapine for preventing nausea and vomiting induced by MEC. The study conducted by Jeon also observed superior efficacy of olanzapine in controlling nausea, with a total TP rate in the olanzapine group significantly higher than the control group (44.0% vs 17.2%; *P* = 0.032), albeit lower than the present study (66.7% vs 33.3%; *P* = 0.003). The Korean study, with its small sample size and lack of stratification for various factors, may have limitations in generalizability and comprehensiveness.

In this study, a relatively small proportion of patients, specifically 5.9% (6/102), received an irinotecan-containing regimen. A study conducted by Paul et al. addressed the incidence of CINV in patients diagnosed with colorectal cancer undergoing irinotecan chemotherapy, with a group size of 44 patients. Their findings indicated that the administration of a 5-HT3 antagonist and dexamethasone before irinotecan infusion effectively controlled nausea and vomiting within 24 h of chemotherapy, yielding a CR of 86%. Remarkably, most patients (82%) did not experience delayed vomiting, and there was no need for rescue antiemetics. Additionally, the study indicated that routine prevention of delayed CINV after irinotecan use may have limited value (Hesketh et al. 2011). Given the limited representation of patients receiving irinotecan-containing regimens in the current study, further investigation is warranted to ascertain the efficacy of olanzapine for preventing CINV in this specific population.

Regarding the enhancement of quality of life, a statistically significant difference was observed in the Functional Living Index-Emesis (FLIE) index related to nausea during the total phase in the olanzapine group when compared to the control group in this study (*P* = 0.009). Furthermore, in elderly women, the FLIE index on nausea in the olanzapine group demonstrated a trend towards enhancement compared to the control group (*P* = 0.089). In both age groups, the olanzapine group exhibited higher proportions of patients with FLIE scale scores surpassing 108, with values of 84.31% vs 58.8% (*P* = 0.009) and 84.38% vs 58.33% (*P* = 0.041), respectively, indicative of superior nausea control. Similar conclusions were reached in the studies by Jeon and Wu, reinforcing the appropriateness of using the TP rate as the primary research index in this study (Jeon et al. 2019; Wu et al. 2020a). Regarding chemotherapy toxicities, a significant amelioration in appetite loss was observed in the olanzapine group when compared to the control group (43.13% vs. 27.45%; *P* = 0.036), consistent with findings in previous studies demonstrating the ability of olanzapine to enhance the appetite of patients (Mizukami et al. 2014; Decker et al. 2006). In terms of adverse reactions, the primary adverse reaction in the olanzapine group in this study was drowsiness, with a higher incidence compared to the study by Wu (52.94% vs 47.5%). This discrepancy may be attributed to the older mean age (> 60 years) of patients in the present study, aligning with evidence that the sedative effects of olanzapine tend to be more pronounced in older individuals (Lei et al. 2017). However, it is important to acknowledge the limitations of this study, including the relatively small sample size and the omission of potential risk factors for analysis, such as smoking history, history of motion sickness, and vomiting during pregnancy. Notably, emerging studies indicate potential associations between genetic polymorphisms and chemotherapy-induced nausea and vomiting (Jin et al. 2023), implying that individual risk factors could influence the efficacy of CINV prevention. The absence of significant differences in CR rates observed in this study might be influenced by these individual risk factors. As genetic testing technology advances and becomes more cost-effective, further exploration of these associations may enhance our understanding of CINV prevention efficacy.

In conclusion, the results of this study affirm that olanzapine effectively mitigates CINV induced by MEC regimens in female patients across all age groups, including the elderly. Olanzapine not only enhances appetite but also contributes to an enhanced quality of life for these patients. Existing research has indicated that a daily dose of 2.5 mg of olanzapine is adequate for preventing CINV (Bun et al. 2018). Future directions may explore the feasibility of sustained low-dose olanzapine administration coupled with patient-initiated reporting for outcome management, providing a promising approach for controlling nausea and enhancing the quality of life during the delayed phase following chemotherapy (Basch et al. 2022).

## Data Availability

The relevant supporting data are available from the author upon request.
